# Water Dynamics in a Peptide-appended Pillar[5]arene Artificial Channel in Lipid and Biomimetic Membranes

**DOI:** 10.3389/fchem.2021.753635

**Published:** 2021-10-29

**Authors:** Daniel Ryan Barden, Harish Vashisth

**Affiliations:** Department of Chemical Engineering, University of New Hampshire, Durham, NH, United States

**Keywords:** artificial water channel, peptide appended pillar[5]arene channel, molecular dynamics, water transport, biomimetic membrane

## Abstract

Peptide-appended Pillar[5]arene (PAP) is an artificial water channel that can be incorporated into lipid and polymeric membranes to achieve high permeability and enhanced selectivity for angstrom-scale separations [Shen et al. *Nat. Commun.* **9**:2294 (2018)]. In comparison to commonly studied rigid carbon nanotubes, PAP channels are conformationally flexible, yet these channels allow a high water permeability [Y. Liu and H. Vashisth *Phys. Chem. Chem. Phys.* **21**:22711 (2019)]. Using molecular dynamics (MD) simulations, we study water dynamics in PAP channels embedded in biological (lipid) and biomimetic (block-copolymer) membranes to probe the effect of the membrane environment on water transport characteristics of PAP channels. We have resolved the free energy surface and local minima for water diffusion within the channel in each type of membrane. We find that water follows single file transport with low free-energy barriers in regions surroundings the central ring of the PAP channel and the single file diffusivity of water correlates with the number of hydrogen bonding sites within the channel, as is known for other sub-nm pore-size synthetic and biological water channels [Horner et al. *Sci. Adv.* **1**:e1400083 (2015)].

## 1 Introduction

Water transport in sub-nm size pores occurs in various structures including carbon nanotubes (CNTs), derivatives of imidazoles and pillararenes, and biological channel proteins (Corry, 2008; Majumder et al., 2005; Tunuguntla et al., 2017; Licsandru et al., 2016; Cheruzel et al., 2003; Hu et al., 2012; Shen et al., 2015; Barboiu and Gilles, 2013; Si et al., 2011; Shen et al., 2015; Grzelakowski et al., 2009; Kumar et al., 2007). Given the limitations in experimental techniques for probing water dynamics in sub-nm pores, computational methods rooted in quantum mechanical (QM) calculations and molecular dynamics (MD) simulations have been employed to probe water structure and transport characteristics (Errington and Debenedetti, 2001; Hummer et al., 2001; Kalra et al., 2003; Andreev et al., 2005; Liu et al., 2005; Giovambattista et al., 2006; Won et al., 2006; Rasaiah et al., 2008; Zhu and Hummer, 2010; Altabet and Debenedetti, 2014; Tunuguntla et al., 2017; Li et al., 2018; Liu and Vashisth, 2019).

**FIGURE 1:**
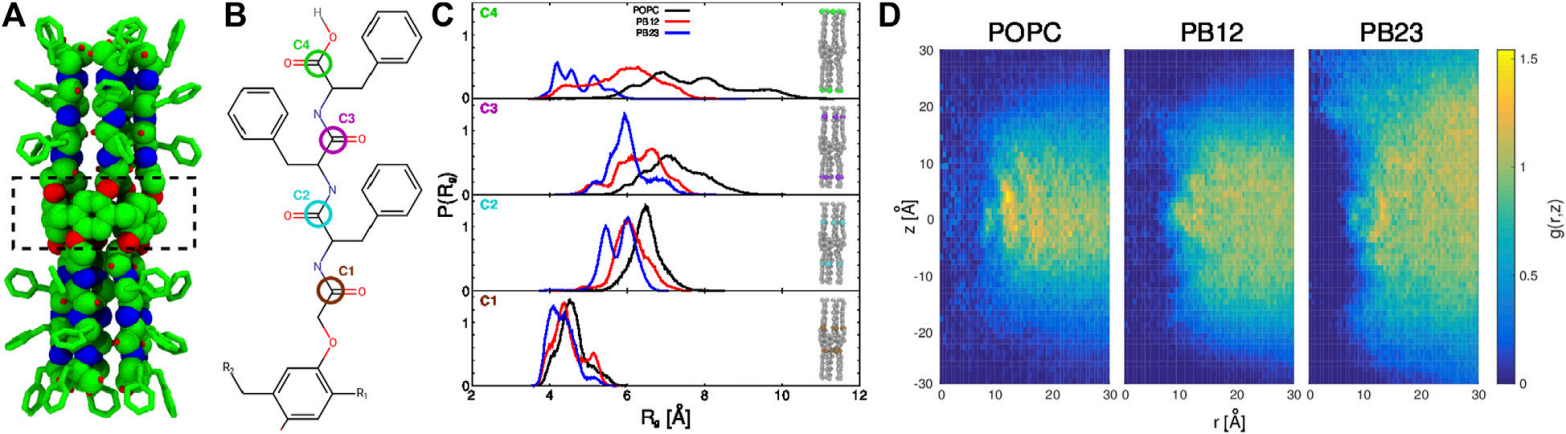
**(A)** The structure of the PAP channel is shown in a space-filling representation (green, carbon atoms; blue, nitrogen atoms; and red, oxygen atoms). The hydrogen atoms are omitted for enhanced visualization of the structure. A dotted rectangle marks the central pillar(5)arene ring. **(B)** The chemical structure of a peptide arm of PAP is shown with the carbonyl carbon positions (C1, C2, C3, and C4) colored and labeled. **(C)** The distributions of the radius of gyration, R_*g*_, of carbonyl carbon atoms are shown for each membrane. **(D)** For each of the three types of membranes, shown are the two-dimensional maps of the radial and axial distribution function, *g* (*r*, *z*) = $\frac{N}{\rho dV}$ , where *N* is the average number of carbon atoms in each bin, *ρ* is the bulk number density of the hydrophobic block, *dV* is the volume of each bin defined as *dV* = *π*[(*r* + *dr*)^2^ − *r* ^2^]*dz* with *dr* and *dz* as the bin-widths along the *r* and *z* directions. The *z*-axis is aligned along the length of the channel and passes through the center of the pillar[5]arene ring of PAP.

While CNTs have been used as a model system for these studies (Hummer et al., 2001; Kalra et al., 2003; Tunuguntla et al., 2017; Li et al., 2018), primarily because they offer an idealized environment due to their relatively rigid as well as chemically and topologically homogeneous structures, synthetic flexible channels based upon pillararene derivatives, in particular peptide appended pillar[5]arene (PAP) water channels, are emerging as novel biomimetic pores (Hu et al., 2012; Shen et al., 2015) to study the interplay of water transport and conformational flexibility of the channel in different membrane environments. As opposed to CNTs, PAP has a flexible and chemically heterogeneous structure with a central pillar[5]arene ring (Figure 1A) of ∼4.5 Å diameter to which are attached 10 flexible peptide arms, 5 on each side of the central ring and each consisting of 3 phenylalanine (Phe) residues. The Phe side-chains interact with the hydrophobic core of the membrane allowing the backbone of the peptide arms to line the interior of the channel, creating a hydrophilic environment on either side of the central ring.

We have previously reported that PAP can be successfully inserted into lipid and block-copolymer (BCP) based membranes with the channel permeability between ∼10^8^–10^9^ water molecules s^−1^ (Shen et al., 2018; Barden and Vashisth, 2018). The biomimetic membranes based on PAP have a channel packing density roughly two orders-of-magnitude higher than the membranes based on CNTs (Shen et al., 2015, 2018). We also reported that the membrane environment (lipid *vs.* BCP) as well as channel-channel interactions affect the flexibility of PAP and its permeability (Shen et al., 2018; Liu and Vashisth, 2019). Therefore, our previous work has quantified macroscopic properties of PAP including its permeability and diffusivity within different membrane environments. However, the thermodynamics and the water transport mechanism as well as the correlation of single-file water transport with the hydrogen bonds in channel-lining residues (Horner et al., 2015) have not been studied in detail for the PAP channel. In this work, we study the dynamics of water molecules within PAP and map the free energy surface of water transport in PAP for different membrane environments, thereby quantifying the effect of membrane on water structure and dynamics, especially single-file water transport.

## 2 Methods

### 2.1 Molecular Dynamics Simulation Setup

We conducted all MD simulations using the NAMD software (Phillips et al., 2005) and prepared as well as analyzed all systems using the VMD software (Humphrey et al., 1996). Consistent with our previous work (Shen et al., 2018; Barden and Vashisth, 2018; Liu and Vashisth, 2019), we used the CHARMM force-field (MacKerell et al., 1998; Huang et al., 2017) for water and lipid molecules, the polyethylene oxide (PEO) chains, and the CHARMM compatible force-field for PAP (Shen et al., 2015) and the polybutadiene (PB) chains (Barden and Vashisth, 2018). For analyses of water dynamics in PAP and to sample initial coordinates of water molecules in the channel for free energy calculations (*vide infra*), we used our previous data (Shen et al., 2018) from triplicate MD simulations (750 ns per membrane) of PAP in each of the three types of membranes (POPC, PB_12_PEO_9_, and PB_23_PEO_16_).

Briefly, in these simulations we used equilibrated all-atom models of POPC (70 Å × 70 Å) and BCP (80 Å × 80 Å) membranes and incorporated a single PAP channel by aligning the center of mass of the membrane as well as that of the channel to the origin (0 0 0). We then equilibrated the channel-embedded membrane configurations in a three-step process involving first fixing of all-atoms in the system except those in the lipid/BCP tails (step 1), followed by fixing only the atoms in the PAP channel while keeping water molecules out (step 2), and finally releasing all constraints for long time-scale MD simulations in the NPT ensemble using a time-step of 2 fs, a temperature of 303 K (controlled via a Langevin thermostat), and a pressure of 1 atm (controlled via a Nos $\overset{´}{e}$ -Hoover barostat).

### 2.2 Free Energy Calculations

We used the single-sweep method (Maragliano and Vanden-Eijnden, 2008) to reconstruct the free energy surface of water transport in PAP for each of the three different types of membranes. This method has been successfully used for efficiently computing multidimensional free energy surfaces (Maragliano et al., 2010; Yu et al., 2015; Mohammadi and Vashisth, 2017; Liu et al., 2018). In this method, the free energy surface is resolved in a set of collective variables (CVs), which are functions of the atomic Cartesian coordinates. Specifically, the mean forces are computed at selected CV positions that are sampled via a conventional MD simulation or using an enhanced sampling MD technique, most commonly temperature-accelerated MD (Maragliano and Vanden-Eijnden, 2006).

A suitable set of CVs used for studying the transport of small molecules (e.g., O_2_, CO, H_2_O) are the Cartesian coordinates of the center of mass of the small molecule (Maragliano et al., 2010; Lapelosa and Abrams, 2013; Yu et al., 2015; Mohammadi and Vashisth, 2017; Liu et al., 2018), which we used to resolve the free energy surfaces of water transport in PAP. It is necessary to choose distinct CV positions (also termed as “centers”) for water molecules that are distributed throughout the PAP channel. For a set of CV positions defined by the chosen N-centers, the reaction coordinate is 3N-dimensional with three Cartesian coordinates per center. We harvested these unique centers for water molecules from our earlier MD simulations of PAP in three types of membranes (Shen et al., 2018). Specifically, we first aligned the atomic coordinates from each MD trajectory to the initial structure of PAP by computing the root mean squared deviation (RMSD) of the carbon atoms in the central pillararene ring of PAP. We then extracted the coordinates of all water molecules restricted to a cylindrical channel volume (measured by a cylinder of radius *r* × height *h*, centered around the pillar[5]arene ring of PAP) for each type of membrane: 12 Å × 12 Å (POPC and PB_12_PEO_9_) and 12 Å × 16 Å (PB_23_PEO_16_). Out of all water positions sampled within this cylindrical volume, we chose 369 (POPC), 428 (PB_12_PEO_9_), and 230 (PB_23_PEO_16_) unique centers (Supplementary Figure S1) for each system so that each water molecule chosen was at least 1 Å away from neighboring water molecules.

For each system, we then computed the mean-force at each center (*f*_*k*_) via a 1 ns long MD simulation by harmonically restraining (with a spring constant of *κ* = 10 kcal/mol⋅Å^2^) the CV-value for each water molecule at the chosen center. We also applied weak restraints (with a spring constant of *κ* = 2 kcal/mol⋅Å^2^) on each carbon atom of the central pillar[5]arene ring to make each system rototranslationally invariant. Therefore, we conducted 369 (POPC), 428 (PB_12_PEO_9_), and 230 (PB_23_PEO_16_) new MD simulations, one restrained MD simulation per center. Consistent with earlier studies (Mohammadi and Vashisth, 2017; Liu et al., 2018), we observed the convergence of mean forces for most centers in short 1 ns MD simulations; for any centers where the mean forces were not converged within 1 ns time-scale, we simulated for an additional 1 ns until the convergence of the mean force was observed (always within 5 ns for any center).

Using the converged mean force at each center (*f*_*k*_) in all systems, we globally reconstructed the free-energy surface *A* (*z*) (Eq. 1) as a linear combination of Gaussian radial basis functions (*ϕ*_*σ*_, where *σ* is the Gaussian width). The free energy functional *A* (*z*) is given as below:

$$A\left(z\right)=\overset{K}{\underset{k=1}{\sum }}a_{k}\phi_{\sigma }\left(\left|z-z_{k}\right|\right)+C$$ (1)

where *a*_*k*_ is the *k*th coefficient in the expansion, *K* is the total number of centers, and *C* is a constant for adjusting the height of *A*(*z*). We obtained the optimized values of all *a*_*k*_ and *σ* via a least square fitting procedure based on Eq. 2 (Maragliano and Vanden-Eijnden, 2008; Monteferrante et al., 2009)

$$E\left(a,\sigma \right)=\overset{K}{\underset{k=1}{\sum }}{\left|\overset{K}{\underset{k^{\prime }=1}{\sum }}a_{k^{\prime }}\nabla_{z}\phi_{\sigma }\left(\left|z_{k}-z_{k^{\prime }}\right|\right)+f\left(z_{k}\right)\right|}^{2}$$ (2)

The optimized values of *σ* for each system were 2.0 Å (POPC), 1.6 Å (PB_12_PEO_9_), and 2.3 Å (PB_23_PEO_16_), and the relative residual ( ${\left(E\left(a,\sigma \right)/\sum_{k}|f_{k}{|}^{2}\right)}^{1/2}$ ) values for each system were 0.63 (POPC), 0.63 (PB_12_PEO_9_), and 0.64 (PB_23_PEO_16_). The reconstructed free energy surfaces facilitate identification of free energy minima as locations between which water molecules shuttle for transport through the channel.

## 3 Results and Discussion

### 3.1 Peptide Appended Pillar[5]arene Conformational Dynamics and Membrane Reorganization

In our previous work (Shen et al., 2018), we highlighted that the overall structure of the PAP channel is more flexible within the POPC membrane in comparison to the BCP based membranes (PB_12_PEO_9_ and PB_23_PEO_16_). We also observed that the PAP channel stably maintains its orientation (at ∼15–20° relative to the membrane normal) in each membrane although the height of the PAP channel (∼4.0 nm) differs from the hydrophobic thickness of three membranes (∼3.7 nm, POPC; ∼5.1 nm PB_12_PEO_9_, and ∼6.0 nm, PB_23_PEO_16_). To further expand on these observations, we first quantify instrinsic conformational changes in PAP in each of the three types of membranes. Specifically, we measured the radius of gyration (R_g_) of four carbonyl carbon atoms at different positions along the peptide backbone (labeled C1, C2, C3, and C4 in Figure 1B). These data show an increase in the width of R_g_ distributions (Figure 1C) for the carbonyl carbon atoms located away from the central ring of the PAP channel in each membrane. This is consistent with the structure of the PAP channel where the peptide arms are only connected at the central ring and are otherwise flexible.

The distributions show that the mean value of R_g_ (⟨R_*g*_⟩) increases in all membranes as one moves from the C1 to C2 carbon atoms, but from the C2 to C3 carbon atoms, there is a smaller increase in ⟨R_*g*_⟩ in POPC and PB_12_PEO_9_ membranes, while no appreciable change in the PB_23_PEO_16_ membrane. When comparing the C3 and C4 carbon positions, there is no appreciable change in ⟨R_*g*_⟩ among POPC and PB_12_PEO_9_ membranes, but there is a decrease in the PB_23_PEO_16_ membrane. This implies that in the PB_23_PEO_16_ membrane, instead of blooming open like a cone, the PAP channel adopts a conformation with constriction points at the ends similar in size to central ring with two larger diameter pockets on either side of the central ring. The R_g_ distributions also indicate that the C4 carbon position is more flexible in the POPC membrane, followed by in the PB_12_PEO_9_ and PB_23_PEO_16_ membranes.

We further observed that the PAP channel affects the structure of the surrounding membrane. To characterize this, we have computed the two-dimensional radial and axial distribution function *g* (*r*, *z*) (Figure 1D), as has been done to analyze the structure of lipids surrounding CNTs along the radial coordinate (Vögele et al., 2018). We chose the axial as well as the radial coordinate because the structure of the PAP channel is flexible unlike the symmetric and rigid structure of CNTs. Similar to the lipid structure around CNTs (Vögele et al., 2018), we observed well-ordered peaks for lipids in the POPC membrane showing their ordered organization around the PAP channel, while in BCP membranes we observed scattered peaks, lacking regularity, indicating that the polymer chains in BCP membranes structure themselves around PAP differently than do lipids in the POPC membrane. The *g* (*r*, *z*) for the PB_23_PEO_16_ membrane also shows the adaptation of these thicker membranes to the height of the PAP channel, consistent with our previous work (Shen et al., 2018).

### 3.2 Water Structure and Dynamics in Peptide Appended Pillar[5]arene

**FIGURE 2:**
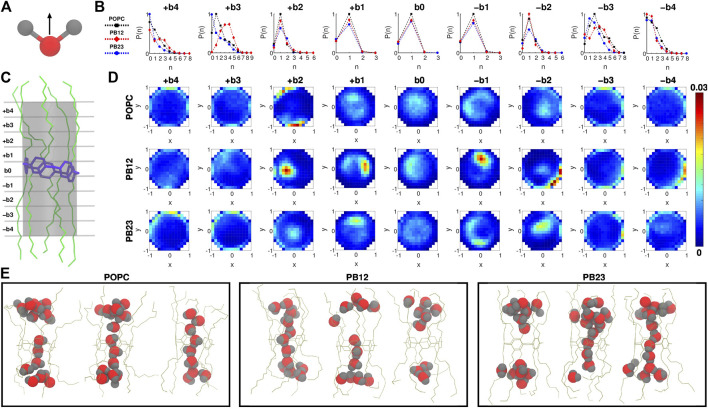
**(A)** The direction vector along the dipole moment of a water molecule is highlighted. **(B)** Probability of finding “n” water molecules, P (n), in each of the 9 bins is shown. **(C)** A snapshot highlighting the 9 bins partitioning the channel, where b_0_ is the bin spanning the central pillar[5]arene ring of the channel, and + b_*i*_ and − b_*i*_ (*i* = 1, 2, 3, and 4) are bins above and below the central ring. **(D)** Two dimensional histograms highlighting the water orientation via *x*-*y* plane projections of the dipole vector of water molecules. **(E)** Snapshots highlighting water structure within PAP in each membrane.

To investigate water orientation (Figure 2A) and dynamics within the PAP channel for each of the three membranes, we partitioned the channel structure into nine sections (i.e., bins b_0_, ±b_1_, ±b_2_, ±b_3_, and ±b_4_; Figure 2C) where each section/bin is defined by a cylinder of 4.5 Å in diameter and 2.5 Å in height. Within each bin, we computed the probability of finding “n” water molecules [P (n); Figure 2B] and the orientation of water molecules (Figures 2A,D). We observed that the probability of finding a single water molecule, i.e. P (1), is significant within the bin b_0_ (centered on the pillar[5]arene ring) as well as in neighboring bins on both sides ± b_1_ (Figure 2B), while the probability of finding no water molecules P (0) is small and of finding more than a single water molecule [e.g., P (2), P (3)] is negligible. This implies that the PAP channel may occasionally experience a dewetting transition, as observed in other channels (Beckstein and Sansom, 2003, 2004; Sotomayor and Schulten, 2004; Liu et al., 2005; Thomas et al., 2014) and in our earlier work on PAP (Liu et al., 2018), but it is highly likely that a single water molecule is always present within the central pillar[5]arene ring.

The likelihood of observing a single water molecule within the central-ring region and in areas immediately surrounding it indicates single file mechanism of water transport (as highlighted in snapshots showing the water structure; Figure 2E) within the relatively rigid structural parts of PAP (i.e., bins b_0_ and ±b_1_). For the next bins±b_2_, we still observe a higher probability of observing a single water molecule although there is a finite probability of finding a second water molecule and a negligible probability of finding a third water molecule. The likelihood of observing more than two water molecules is higher in bins ± b_3_. However, moving to areas of the PAP channel with a significantly higher flexibility (bins±b_4_), we find a higher probability of having few water molecules and a lower probability of having more than two water molecules, which means that the flexibility of the channel at the termini of peptide arms hinders water transport, as has been pointed out in studies of other systems (Andreev et al., 2005; Liu et al., 2005; Zhu and Hummer, 2010; Altabet and Debenedetti, 2014).

We also report the orientation of water molecules within each bin for all three membranes (Figure 2D). Specifically, we normalized the vector pointing along the dipole moment of a water molecule (Figure 2A) and computed the projection of the normalized vector on the *x*-*y* plane (for reference, the *z*-coordinate is aligned with the axis of the PAP channel) (Figure 2D). Water molecules oriented with the normalized vector parallel to the *x*-*y* plane will have points projected near the edges of a unit circle, while if oriented perpendicular will have points projected near the origin or the center of the unit circle. In the outermost bins (±b_3_ and ±b_4_), we observed a higher density of dipole vector projections concentrated along the edges of the circle indicating that water molecules are oriented parallel to the *x*-*y* plane and interact with the peptide arms via hydrogen bonds. A previous study of sub-nanometer confinement of water by hydrophilic surfaces showed that hydrogen bonding to the surface, as we also observe in the PAP channel, may increase the viscosity of water by seven orders of magnitude (Major et al., 2006). We further report the average residence time of water molecules in each bin (Supplementary Figure S2) which ranges between ∼30 ps and ∼110 ps.

A transition toward a water wire appears around bins±b_2_. These bins correspond to the more rigid C1 carbonyl carbon region (Figure 1C) and demarcate the location where the probability of finding more than two water molecules becomes negligible (Figure 2B). This region is also still relatively hydrophilic, indicating that a water wire formed near the central ring will be a dominant structural feature over a length equivalent to ∼3 water molecules, with 1 water molecule each spanning bins −b_1_, b_0_, +b_1_. This behavior is consistently observed in all three membrane types. Moreover, water molecules in the interior of the PAP channel appear to partition into states where the hydrogen atoms are pointing up or down (Figure 2D) consistent with previous studies of water movement in narrow pores (Rasaiah et al., 2008; Errington and Debenedetti, 2001; Hummer et al., 2001; Rasaiah et al., 2008; Fumagalli et al., 2018).

**FIGURE 3:**
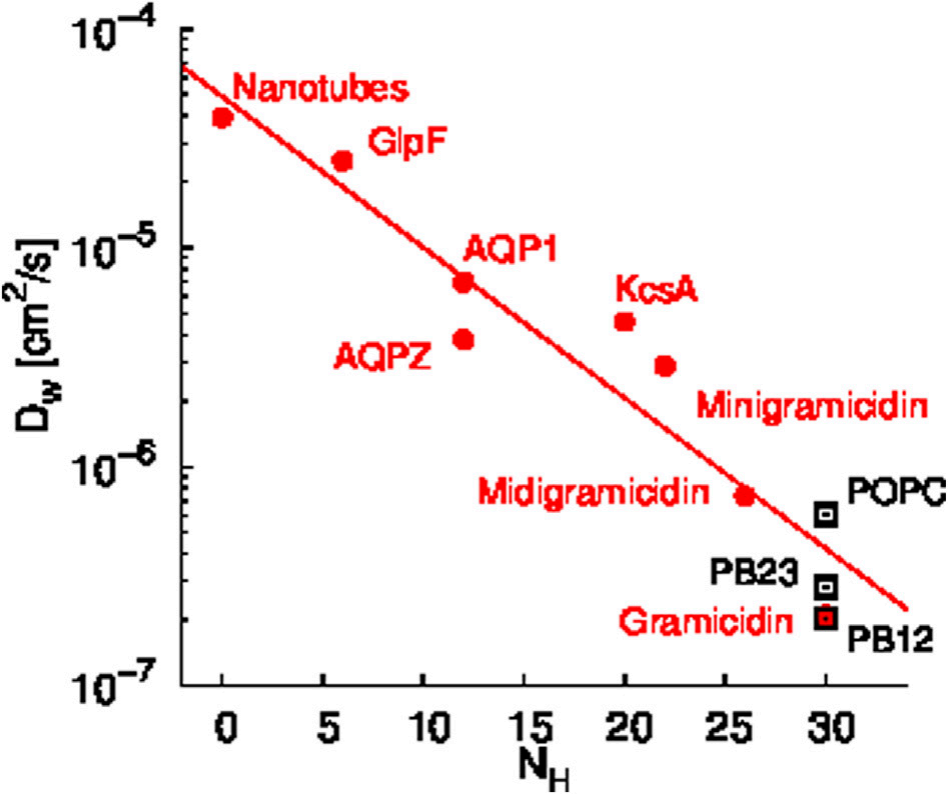
Scaling relationship between the diffusion constant of single file water transport (D_w_) and the number of hydrogen bonding sites in a given channel (N_*H*_) is shown. Data shown in red circles are from previous studies of several synthetic and biological water channels (Hoomann et al., 2013; Horner et al., 2015; Berezhkovskii and Hummer, 2002; Saparov et al., 2006) and data shown in black rectangles are for PAP in each membrane. The D_*w*_ values for PAP were computed based on permeability measurements reported in our earlier work (Shen et al., 2018).

A scaling relationship between the diffusion constant of single file water transport (D_*w*_) and the number of hydrogen bonding sites in a given channel (N_*H*_) has been shown by Horner et al. (Horner et al., 2015). Since we observed single file water transport within the PAP channel, we tested whether the water transport in PAP follows this scaling law. Specifically, we estimated that there are approximately 30 hydrogen bonding sites in the regions of single file transport in PAP and we computed D_w_ for water transport in PAP for each of the three membranes. We found that the D_w_ *vs.* N_*H*_ trend for water transport in PAP follows the scaling trend (Figure 3) observed in other single file water transport channels (Hoomann et al., 2013; Horner et al., 2015; Berezhkovskii and Hummer, 2002; Saparov et al., 2006). Horner et al. (Horner and Pohl, 2018) have also shown that a Gibbs activation energy barrier ( $\Delta G_{t}^{‡}$ ) of ∼5 kcal/mol is hallmark of water movement through a protein water channel and that the activation energy can be measured based on the permeability of a given protein channel. Based on our previous measurements of water permeability in the PAP channel (Shen et al., 2018) and using the relationship suggested by Horner et al. (Horner and Pohl, 2018), we estimated $\Delta G_{t}^{‡}$ for the PAP channel to range between ∼5.2–5.9 kcal/mol. Additionally, we resolved the three-dimensional free energy surface of water transport in PAP in each membrane, as described below.

### 3.3 Thermodynamics of Water Transport in PAP Channel

The free energy landscape of water transport in narrow pores has been traditionally characterized by pulling a water molecule along a one-dimensional reaction coordinate typically spanning the channel-axis, measuring the pulling forces, and quantifying the free energy barriers (Dudko et al., 2006; Hummer and Szabo, 2001; Park et al., 2003; Won et al., 2006). This method is effective in rigid channels including CNTs because there is little variation in the diameter of the channel and water is restricted to a single one dimensional pathway for transport in a sufficiently narrow space. However, for mapping energy barriers in flexible channels with topological variation (e.g., PAP), a one dimensional reaction coordinate offers a narrow sampling of pathways. Therefore, we applied advanced sampling methods (see Section 2.2) that allow reconstruction of a multidimensional free energy surface for water transport in PAP. Specifically, we applied the single sweep method (Maragliano and Vanden-Eijnden, 2008; Monteferrante et al., 2009) to reconstruct the three-dimensional free energy surface of water transport in PAP for each membrane.

**FIGURE 4:**
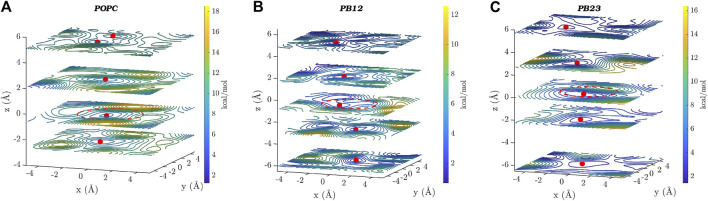
Two dimensional (*x*–*y*) projections of the 3D free energy surface of water transport are shown at different *z*-values for each of the three membranes: **(A)** POPC, **(B)** PB_12_PEO_9_, and **(C)** PB_23_PEO_16_. Several free-energy minima are marked with a red point on two-dimensional projections of the free energy surface. A red circle with a dotted line is drawn to indicate the approximate size and the location of the central ring of the PAP channel. The free-energy scale is indicated by the color palette.

In Figure 4, we show two dimensional (*x*-*y* plane) slices of the three dimensional free energy surface at different *z*-values, where the *x*-*y* plane is parallel to the central ring (located near *z* = 0 and marked by a dotted red circle) of the PAP channel and the *z*-axis is aligned along the vector through the origin and perpendicular to the plane of the central ring. Consistent with the observation that there is a higher likelihood of finding water molecules within the central ring of PAP and areas immediately surrounding it, we observed three free energy minima (marked by red spheres in Figure 4) within PAP, one minimum near the central ring (*z* = 0) and two minima around *z* = ±2.0/2.5 Å, for each membrane. The free-energy barriers for water hopping between the minima around the central ring are below or around ∼2 kcal/mol, indicating single file water transport with low free energy barriers.

In addition to the free energy minima around the central ring, we also observed additional free energy minima within PAP in structural regions away from the central ring and near the peptide arms. The location of free energy minima on both sides of the central ring for each membrane and among different membranes is distinct, further confirming the asymmetry and unique conformations of the flexible peptide arms of the channel in each membrane. Moreover, one-dimensional projections of the 3D free-energy surfaces (Supplementary Figure S3) do not fully capture all other minima located on either side of the ring, which highlights the usefulness of a 3D free-energy surface reconstruction in visualizing these minima. We also observed regions of higher free energy in membrane areas surrounding the channel indicating that water is likely confined within the PAP channel and unlikely to leak through the peptide arms or around the channel (Shen et al., 2018). However, it is noteworthy that a different structural variant of the PAP channel, termed PAH[4] made up of hybrid[4]arenes with a ∼3 Å pore-size, was shown to transport water molecules via an entirely different mechanism due to its distinct structural architecture, where water transport can occur outside of the central pore (Song et al., 2020). Our results collectively show that the water structure and dynamics in the rigid structural parts of the PAP channel are consistent with the single file water transport with low free energy barriers, as observed in several other narrow pores including CNTs, while the water structure near the flexible peptide arms is affected by unique conformational dynamics of the channel in lipid and polymeric membranes.

## 4 Conclusion

We have studied water structure and dynamics in a sub-nm (∼4.5 Å) pore-size artificial PAP water channel which can be successfully incorporated in lipid and polymeric membranes (Shen et al., 2018; Barden and Vashisth, 2018; Liu and Vashisth, 2019). We highlight conformational changes in the PAP structure for each membrane and found that the structure of the channel is relatively rigid near the central pillar[5]arene ring and in the regions surrounding it although significant conformational flexibility is observed in regions of the peptide arms away from the central ring. We observed an ordered structure of lipids surrounding the channel, but a less ordered structure of chains in the polymeric membranes, which allows thicker polymeric membranes to adapt to the height of the channel. In the rigid parts of the channel, we found single file water transport with low free energy barriers, where the hydrogen atoms of water molecules are pointing upward or downward to align along the channel axis during water permeation events through the central ring of the channel. We determined that the water permeability of the channel is consistent with the Gibbs activation energy barrier for single-file water transport (Horner and Pohl, 2018), which also follows the scaling law relating water diffusivity with hydrogen-bonding sites within other transport channels (Horner et al., 2015).

## Data Availability

The original contributions presented in the study are included in the article/Supplementary Material, further inquiries can be directed to the corresponding author.
