# Supplementary material for: Water Dynamics in a Peptide-appended Pillar[5]arene Artificial Channel in Lipid and Biomimetic Membranes
Source: Front Chem. 2021 Oct 29;9:753635. doi: 10.3389/fchem.2021.753635 (PMC8586425; doi:10.3389/fchem.2021.753635)
Supplement: Supplementary file 1 [file DataSheet1.PDF]

Water Dynamics in a Peptide-appended Pillar[5]arene Artificial Channel in Lipid and Biomimetic Membranes

Daniel Ryan Barden,[1] and **Harish Vashisth**[2,*]
[1] *Aspen Technologies, Inc., Bedford, MA 01730, USA*
[2] *Department of Chemical Engineering, University of New Hampshire, 33 Academic Way, Durham, NH 03824, USA*

Correspondence*: harish.vashisth@unh.edu

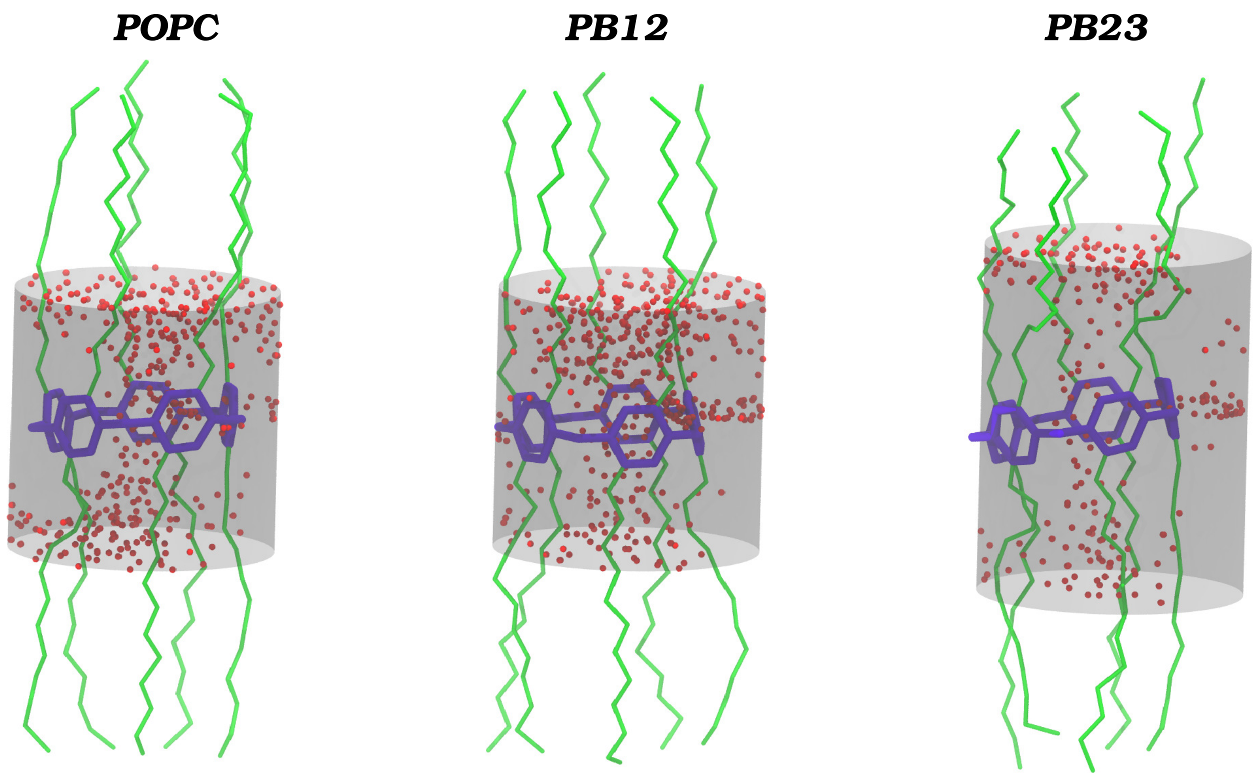

Figure S1. Shown are the centers (red spheres) representing unique positions of water molecules used in single-sweep free energy reconstruction: 369 centers (POPC), 428 centers ($PB_{12}PEO_9$), and 230 centers ($PB_{23}PEO_{16}$). The backbone of the PAP channel and the central pillar[5]arene ring are shown in green and violet stick representations, respectively. The channel volumes used to extract coordinates of water molecules and to choose unique water centers for each membrane are shown as gray cylinders.

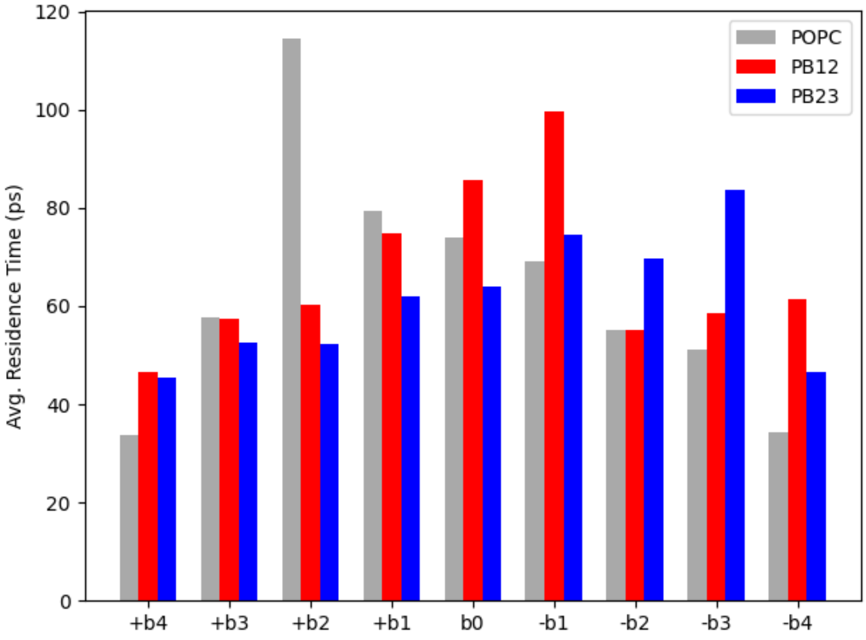

Figure S2. The average residence time (ps) of water molecules in each bin (cf. Fig 2C) of the PAP channel for each membrane are shown. All water molecules that entered the channel, irrespective of their net transport across the channel, contributed to the residence time.

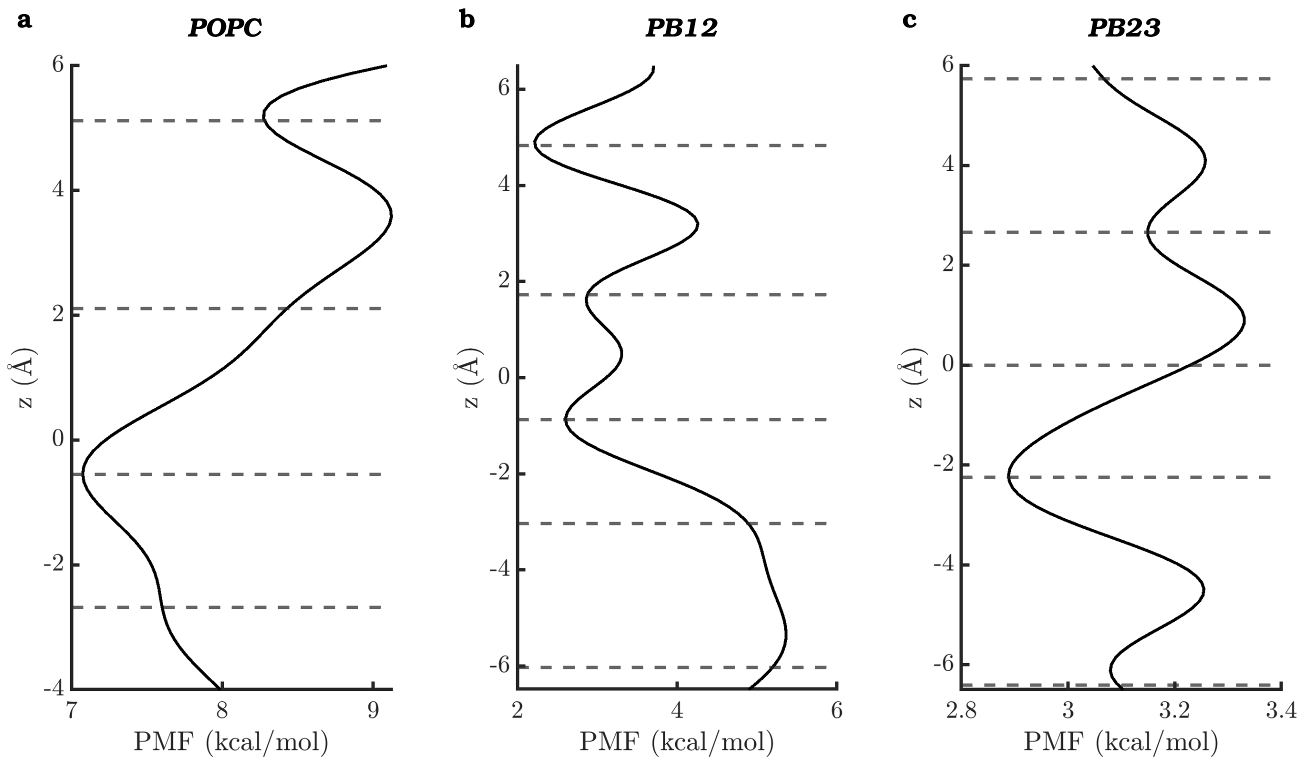

Figure S3. One dimensional potential of mean force (PMF) projections of the 3D free energy surfaces (cf. Fig. 4) are shown for each membrane. The projections are based on the z-axis passing through the free-energy minimum nearest to the $z = 0$ plane in each membrane. It is noteworthy from one dimensional PMFs that other key minima for water transport, especially those located on either side of the central pillar[5]arene ring of PAP, are not aligned along the z-axis passing near the center of the channel. This observation highlights the usefulness of resolving 3D free energy surfaces (Fig. 4) for discovering additional free energy minima in conformationally flexible water channels (e.g., PAP).
